# Identification and Characterization of Transcripts Regulated by Circadian Alternative Polyadenylation in Mouse Liver

**DOI:** 10.1534/g3.118.200559

**Published:** 2018-09-04

**Authors:** Kerry L. Gendreau, Benjamin A. Unruh, Chuanli Zhou, Shihoko Kojima

**Affiliations:** * Department of Biological Sciences; † Biocomplexity Institute, Virginia Tech, Blacksburg, VA, 24061

**Keywords:** circadian rhythms, alternative polyadenylation, *in silico*, mouse liver

## Abstract

Dynamic control of gene expression is a hallmark of the circadian system. In mouse liver, approximately 5–20% of RNAs are expressed rhythmically, and over 50% of mouse genes are rhythmically expressed in at least one tissue. Recent genome-wide analyses unveiled that, in addition to rhythmic transcription, various post-transcriptional mechanisms play crucial roles in driving rhythmic gene expression. Alternative polyadenylation (APA) is an emerging post-transcriptional mechanism that changes the 3′-ends of transcripts by alternating poly(A) site usage. APA can thus result in changes in RNA processing, such as mRNA localization, stability, translation efficiency, and sometimes even in the localization of the encoded protein. It remains unclear, however, if and how APA is regulated by the circadian clock. To address this, we used an *in silico* approach and demonstrated in mouse liver that 57.4% of expressed genes undergo APA and each gene has 2.53 poly(A) sites on average. Among all expressed genes, 2.9% of genes alternate their poly(A) site usage with a circadian (*i.e.*, approximately 24 hr) period. APA transcripts use distal sites with canonical poly(A) signals (PASs) more frequently; however, circadian APA transcripts exhibit less distinct usage preference between proximal and distal sites and use proximal sites more frequently. Circadian APA transcripts also harbor longer 3′UTRs, making them more susceptible to post-transcriptional regulation. Overall, our study serves as a platform to ultimately understand the mechanisms of circadian APA regulation.

Post-transcriptional regulation is an integral part of controlling gene expression and can influence when, where, and how much protein will be generated (Keene 2010). The mammalian molecular clock system utilizes multiple post-transcriptional mechanisms, including alternative splicing, mRNA stability, translation, exosome, and miRNA regulation, to drive robust circadian gene expression (Kojima and Green 2015; Green 2017). Genome-wide studies demonstrated that approximately 5–20% of RNAs are expressed rhythmically in mouse liver, and over 50% of mouse genes are rhythmically expressed in at least one tissue, even though these percentages vary among studies due to the difference in experimental conditions and statistical analyses in detecting rhythmicity (Duffield 2003; Fang *et al.* 2014; Zhang *et al.* 2014; Janich *et al.* 2015; Kojima and Green 2015; Sato *et al.* 2017; Guan *et al.* 2018). These rhythmic gene expressions were thought to be driven primarily by rhythmic transcription; however, more recent transcriptome studies challenged the current belief and highlighted the importance of post-transcriptional regulation. For example, approximately 80% of all rhythmically expressed RNAs do not undergo rhythmic *de novo* transcription. Moreover, oscillating mRNA levels are not a prerequisite for rhythmic protein accumulation (Reddy *et al.* 2006; Koike *et al.* 2012; Menet *et al.* 2012; Mauvoisin *et al.* 2014; Robles *et al.* 2014). These studies clearly emphasize that it is not just transcription, but, rather, an intricate interplay between transcriptional and post-transcriptional regulation that is required to generate rhythmicity in gene expression.

One important mechanism of post-transcriptional regulation is the control of poly(A) tails. Despite the traditional view that poly(A) tail formation is a static and non-regulatory process, recent studies clearly demonstrated that poly(A) tails undergo dynamic regulation both in length and location to, ultimately, alter the fate of mRNAs. We and others identified mRNAs with rhythms in their poly(A) tail length (Robinson *et al.* 1988; Gerstner *et al.* 2012; Kojima *et al.* 2012) and demonstrated that this rhythmicity correlates with protein accumulation (Kojima *et al.* 2012). Differential usage of poly(A) sites, an event termed alternative polyadenylation (APA), yields transcripts differing in their 3′-ends, and can impact downstream RNA processing, such as mRNA stability (Mayr and Bartel 2009; Gupta *et al.* 2014), translation (Mayr and Bartel 2009; Lau *et al.* 2010; Pinto *et al.* 2011; Gruber *et al.* 2014; Masamha *et al.* 2014), subcellular localization of mRNAs (An *et al.* 2008; Djebali *et al.* 2012; Neve *et al.* 2016), or even localization of the encoded protein (Berkovits and Mayr 2015). APA regulation is widespread and prevalent, affecting ∼50% of genes in mammals as well as in other taxa, such as plants and flies (Tian *et al.* 2005; Tian and Manley 2016).

APA occurs in response to a variety of extracellular stimuli (An *et al.* 2008; Chang *et al.* 2015), changes in physiological conditions (Takagaki *et al.* 1996; Takagaki and Manley 1998; Sandberg *et al.* 2008; Wang *et al.* 2008; Ji *et al.* 2009; Ji and Tian 2009; Shepard *et al.* 2011), or pathological conditions (Mayr and Bartel 2009; Singh *et al.* 2009). It remains elusive, however, whether the circadian clock system also regulates poly(A) site locations. Interestingly, the protein levels of a few components of the 3′-end processing machinery, such as CSTF77 (gene name: CSTF3) or NUDT21 (gene name: CPSF5), are rhythmic in mouse liver (Mauvoisin *et al.* 2014; Robles *et al.* 2014), raising the possibility that APA is under circadian regulation. In fact, statistical analyses using circadian microarray datasets indicated that APA is under the circadian regulation in mouse white fat and liver (Liu *et al.* 2013; Ptitsyna *et al.* 2017). Although microarrays and RNA-seq both effectively quantify transcriptional levels and the results are reasonably correlated between the two technologies, RNA-Seq outperforms microarrays (even tiling arrays) in detecting transcripts with low abundance, distinguishing various isoforms, and enabling the detection of differentially expressed genes with higher fold-change (Agarwal *et al.* 2010; Zhao *et al.* 2014).

By using circadian RNA-seq datasets in mouse liver, here we report that 57.4% of all genes expressed undergo APA with, on average, 2.53 alternative polyadenylated transcripts per gene. Notably, 2.9% of all expressed genes (or 4.5% of genes undergoing APA regulation) alternate their poly(A) site usage with a circadian period. Distal poly(A) sites and canonical poly(A) signals (PASs) are more frequently used among all APA genes. This was also the case among circadian APA genes; however, rhythmic APA genes have less distinct differences in poly(A) site strength between proximal and distal sites and use proximal sites more frequently. In addition, circadian APA transcripts displayed longer 3′UTRs, which are generally rich in *cis*-regulatory elements, increasing the opportunity to be subjected to post-transcriptional regulation. These characteristics among circadian APA genes will ultimately help us understand the mechanisms of circadian APA regulation.

## Materials and Methods

### Alignment of short reads

Short read files from liver RNA-seq datasets were downloaded from the NCBI SRA database projects: SRP036186 in FASTQ format (Zhang *et al.* 2014), SRP014751 (Koike *et al.* 2012) and SRA025656 (Vollmers *et al.* 2012) in CSFASTA format with QUAL files. All reads were aligned to the mm9 genome using TopHat version 1.3.3 and Bowtie version 0.12.7 (Trapnell *et al.* 2009). To maximize the efficiency of mapping to the transcripts, rather than the genomes, we used a guide GFF file of *M. musculus* (-G option in TopHat) compromising the 3′-ends of all transcripts in mouse tissues (Hoque *et al.* 2013). Reads that were mapped to multiple regions were discarded. Single end SOLiD colorspace reads were aligned with the following options: -Q –C –-library-type fr-second strand –G TandemUTR_ALE.mm9.gff3. Paired-end Illumina reads were aligned with the following options: -r 200–library-type fr-firststrand –G TandemUTR_ALE.mm9.gff3. Read mapping rates were ∼61–65% for the dataset produced from the Illumina platform (Zhang *et al.* 2014), while these from the SOLiD platform (Koike *et al.* 2012; Vollmers *et al.* 2012) were considerably lower (∼21–30% and ∼16–20%, respectively). This may be due to high ribosomal RNA content in the library preparations and/or the inherent problems that arise from converting the SOLiD sequence data file from colorspace to basespace, which is known to have high error rates (Ondov *et al.* 2008). The resulting BAM files were separated into strand-specific files using SAMtools.

### Quantification of poly(A) site usage frequency by MISO

We applied the Mixture-of-Isoforms probabilistic model (MISO) and calculated “ψ values” of each transcript (Katz *et al.* 2010). To this end, we first discarded genes with <20 reads in their 3′UTRs using the GTF 3′-end annotation files (Hoque *et al.* 2013) with the default settings. For both APA_ALE_ and APA_UTR_, we first eliminated isoforms whose average ψ values for all time points were < 0.1. We also eliminated isoforms whose ψ values were 0 at 2 or more time points for the datasets that have 2 circadian cycles, or at 1 or more time points for the dataset that has only1 circadian cycle. We also eliminated transcripts whose average Transcripts Per Million (TPM) of all time points were < 1 (see next section for RNA quantification methods).

To simplify the subsequent analyses, we then extracted the two transcripts isoforms with the highest ψ numbers (average of all time points) for each gene. If the second highest ψ value was identical among two or more isoforms, we treated each combination (of highest and second highest transcripts) as independent and kept all the combinations. We defined the “APA index” of these combinations by calculating = ψ _proximal_ - ψ _distal_. Therefore, a negative APA index represents transcripts whose distal site usage was more frequent than proximal usage, while a positive APA index represents transcripts whose proximal site usage was more frequent than distal site usage.

### Rhythmicity Detection by MetaCycle

All circadian parameters were detected using MetaCycle with the function meta2d (Wu *et al.* 2016). A period window was set to 20 to 28 hr. The datasets were then filtered for rhythmicity with meta2d output values of *P* < 0.05 and Meta2d rAmp > 0.1 (for APA index) or > 0.01 (for RNA abundance). A slightly lower threshold of *P* < 0.06 and Meta2d rAmp >0.09 was used to detect one transcript (*Ccdc93*) that was represented by all three datasets.

### Gene ontology analysis

Gene ontology (GO) analyses were performed using DAVID (Huang da *et al.* 2009a; Huang da *et al.* 2009b). “Functional Annotation Clustering” feature was used, and the clusters with an Enrichment Score > 1.33 (*i.e.*, equivalent of p-value< 0.05) were listed in Table S2.

### 3′RACE assay

3′- Rapid Amplification of cDNA Ends (3′RACE) assay was performed as previously described elsewhere (Scotto-Lavino *et al.* 2006). Briefly, total RNAs were extracted from mouse liver using TRIZOL (Life Tech), treated with TURBO DNase I (Life Tech) for 30min at 37C, and then 5ug of which was subjected to cDNA synthesis using SuperScript II (Life Tech). To specifically amplify target transcripts, nested PCR was performed for all of the genes. The first PCR was performed using primers Qo and Gene Specific Primer 1 (GSP1), followed by Qi and GSP2 with 1/200 diluted DNA samples from the first PCR. The images shown in Figure 2E are the results of the second PCR. Primer sequences are as follows: QTVN reverse transcription: 5′- CCAGTGAGCAGAGTGACGAGGACTCGAGCTCAAGCTTTTTTTTTTTTTTTTTVN-3′, 3RACE_Qo: 5′- CCAGTGAGCAGAGTGACG-3′, 3RACE_Qi: 5′-GAGGACTCGAGCTCAAGC-3′, Akr1c6_GSP1: 5′- ACTGGAGGTCCATTTTGTGC-3′, Akr1c6_GSP2: 5′- CTTGTGCCAGATGTCACTGC-3′, D930014E17Rik_GSP1: 5′- CCCAGGGACCTATTCTGTCA-3′, D930014E17Rik_GSP2: 5′- GCTCCAGT-GCCTTATCCAAG-3′, Sppl3_GSP1: 5′-AGTTTACCTGGCTTCCTCGC-3′, Sppl3_GSP2: 5′-TGATAGACTTGTGGGCTGTG-3′, Oit3_GSP1: 5′-CTGCAGAGCCAGACACTGAC-3′, Oit3_GSP2: 5′-CCCCTCTAGAACGACTTCCTG-3′

### DATA AVAILABILITY

The authors state that all data necessary for confirming the conclusions presented in the article are represented fully within the article. Supplemental material available at Figshare: https://doi.org/10.25387/g3.6741623.

## Results

### Identification of circadian APA genes

**Figure 1:**
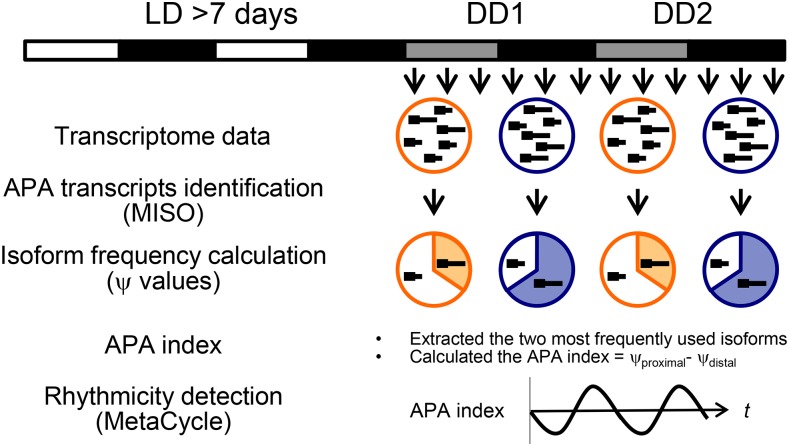
Schematic representation of our strategy to identify circadian APA genes. Briefly, we obtained three circadian transcriptome datasets from NCBI SRA. We then applied the Mixture-of-Isoforms probabilistic model (MISO) to detect alternatively polyadenylated transcripts. MISO further calculated “ψ values” of each transcript that represent the expression levels of alternatively polyadenylated isoforms relative to that of the total transcript within each sample (*i.e.*, each time point). We subsequently extracted isoforms whose ψ numbers (average of all time points) were the two highest to simplify the analyses, and then designated the “APA index” of this combination for each time point, by calculating = ψ _proximal_ - ψ _distal_. The rhythmicity of the APA index was assessed by MetaCycle. White/black bars and gray/black bars indicate light:dark (LD) = 12:12 or constant dark (DD) conditions, respectively, at which tissues were harvested.

Our *in silico* strategy to identify transcripts undergoing circadian APA is described in Figure 1. First, we retrieved circadian transcriptome datasets from the Sequence Read Archive (SRA) at the National Institutes of Health (NIH) (https://www.ncbi.nlm.nih.gov/sra). Three datasets (Koike *et al.* 2012; Vollmers *et al.* 2012; Zhang *et al.* 2014) met our criteria that 1) the samples were harvested in a constant dark condition, 2) the datasets provided strand-specific information, which was critical for the downstream analyses because 3′-ends of transcripts sometimes overlap between genes, and 3) the samples were taken from mouse liver in which many other circadian genome-wide analyses have been performed (*i.e.*, Nascent-seq, GRO-seq, ribosome profiling, proteomics, etc.).

In order to identify APA in the selected datasets, we applied the Mixture of Isoforms Model (MISO) algorithm (Katz *et al.* 2010), a probabilistic framework that identifies differentially regulated isoforms from transcriptome data. MISO was originally intended to detect alternative splicing, but later implemented to detect alternative polyadenylation by incorporating user-defined poly(A) site annotation data (Katz *et al.* 2010; Hooper 2014). We chose MISO over other algorithms, such as DaPars, QAPA, and APAtrap (Xia *et al.* 2014; Ha *et al.* 2018; Ye *et al.* 2018) that identify 3′-ends of transcripts *de novo* and thus require significantly higher read depth. The three circadian transcriptome datasets only had 10-30M mappable and usable reads. Using pre-defined poly(A) sites that had been detected in mouse tissues including liver (Hoque *et al.* 2013), MISO identifies two types of alternatively polyadenylated isoforms: alternative poly(A) sites within 3′UTRs (APA_UTR_), and alternative 3′-terminal exons (ALE) flanking the last intron (APA_ALE_). MISO subsequently quantifies the relative expression levels of alternatively polyadenylated isoforms and returns “ψ values,” which represent the expression levels of alternatively polyadenylated isoforms relative to that of all transcripts at a given time point. The sum of ψ values, therefore, is 1 for each transcript at each time point. We used ψ values as the poly(A) site usage frequency of each alternatively polyadenylated isoform.

From the three mouse liver circadian transcriptomes, MISO detected approximately 5,000 APA_ALE_ and 16,000 APA_UTR_ isoforms that met our filtering criteria (see Materials and Methods), consisting of approximately 8,000 unique genes (4,000 genes for APA_ALE_ and 7,500 genes for APA_UTR_) (Table 1). The average poly(A) sites per gene were 2.53 (Table 1), which is similar to what was observed in other mouse tissues, where it ranged from 1.5 to 4.0 (Tian *et al.* 2005; Hoque *et al.* 2013). Out of the approximately 8000 genes that were expressed, genes that had multiple poly(A) sites represented 57.4% (Table 2). This is also comparable to what has been reported in other mouse tissues, such as retina (49%), EST database (47%), fibroblasts (66%), and cell line mixtures (78.5%) (Tian *et al.* 2005; Hoque *et al.* 2013; Xiao *et al.* 2016; Hu *et al.* 2017).

**Table 1:** Numbers of APA isoforms and genes detected by MISO from mouse transcriptome data

Dataset	APA_ALE_ isoform	APA_UTR_ isoform	Total isoforms	APA_ALE_ unique gene	APA_UTR_ unique gene	Total unique gene	APA_ALE_ per gene	APA_UTR_ per gene	PAS per gene
Dataset1	5,091	16,223	22,124	3,765	7,362	8,171	0.62	1.99	2.61
Dataset2	4,416	13,501	17,917	3,306	6,848	7,668	0.58	1.76	2.34
Dataset3	5,839	18,445	24,284	4,606	8,125	9,174	0.64	2.01	2.65
**Average**	5,115	16,056	21,172	3,892	7,445	8,338	0.61	1.93	2.53

**Table 2:** Numbers of genes/transcripts that possess single or multiple PASs

Dataset	Single PAS genes (ALE)	Single PAS genes (UTR)	Unique Single PAS genes (%)	Multiple PAS transcripts (ALE)	Multiple PAS transcripts (UTR)	Multiple PAS genes (ALE)	Multiple PAS genes (UTR)	Unique Multiple PAS genes (%)
Dataset1	2,499	1,657	3758 (41.3)	3,402	14,566	1,262	4,737	5338 (58.7)
Dataset2	2,492	1,002	3228 (41.0)	1,924	12,499	812	4,198	4645 (59.0)
Dataset3	3,694	2,036	5059 (44.4)	2,145	16,409	912	5,970	6236 (55.6)
**Average**	2,895	1,565	4,015 (42.6)	2,490	14,491	995	4,968	5,406 (57.4)

Using ψ values of each isoform at each time point, we next sought to identify genes that underwent circadian APA regulation. To simplify the statistical analyses and decrease complexity, we focused only on APA_UTR_, which comprised ∼75% of all the APA detected in our analyses (Table 1). We also focused on only the two most frequently used isoforms (*i.e.*, the two transcripts with the highest average ψ values across all time points). This is justified, as the average number of poly(A) sites (including those with a single poly(A) site) was 2.53 (Table 1). We subsequently calculated the “APA index (=ψ _proximal_- ψ _distal_),” which represents the difference in the ψ values between the two APA_UTR_ isoforms. Thus, genes with negative APA indexes use the distal poly(A) site (*i.e.*, produce a longer transcript) more frequently, while genes with positive APA indexes use the proximal poly(A) site (*i.e.*, produce a shorter transcript) more frequently. We then applied the rhythmicity detection algorithm MetaCycle (Wu *et al.* 2016) to detect rhythmic APA events (Figure 1).

**Figure 2:**
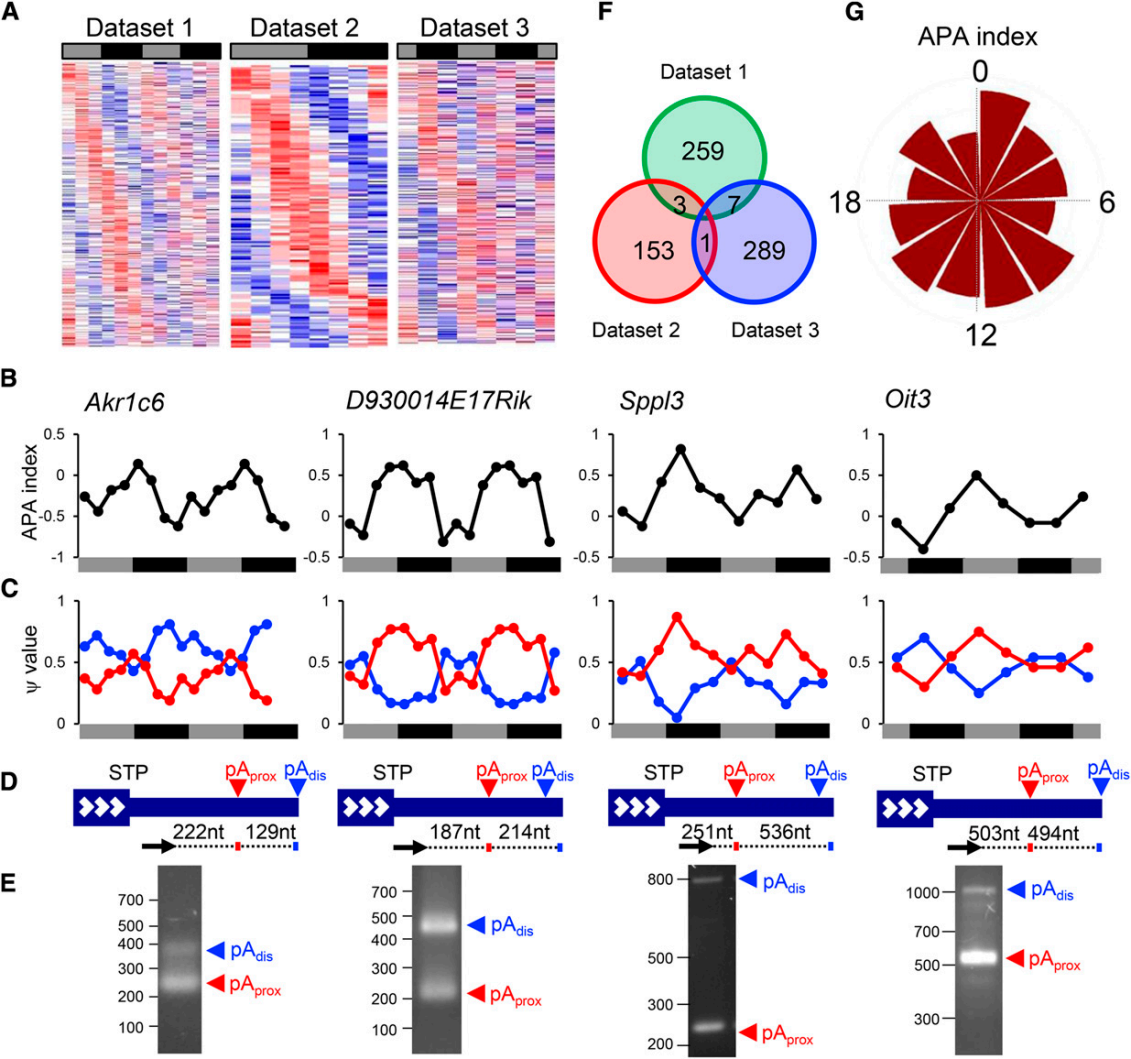
Identification of circadian APA genes. (A) Phase-sorted heat map of the APA index of circadian APA genes. Red and blue colors indicate higher or lower APA index, respectively. Each row represents one gene. (B-C) Examples of circadian APA gene. (B) The APA index profiles (=ψ_proximal_ - ψ_distal_) of *Akr1c6* (Dataset 2), *D930014E17Rik* (Dataset 2), *Sppl3* (Dataset 1), and *Oit3* (Dataset 3) over the circadian cycles. (C) Individual ψ values for transcripts with either proximal (red) and distal (blue) poly(A) site usage over the circadian cycles. Data from Dataset 2 were double-plotted for easier visualization of rhythmicity. (D) 3′-end structures of APA example genes. STP: stop codon, pA_prox_: proximal poly(A) site, pA_dis_: distal poly(A) site, big navy box with white arrowheads: protein-coding region, navy line: 3′UTR. Black arrows represent the locations of the primers used in the 3′RACE assay in (E). (E) 3′RACE assay. Numbers on the left represent the DNA size makers. (F) Venn Diagram of the number of circadian APA transcripts among the three datasets. (G) Circular histogram plots of circadian APA index. Each wedge represents a 2-h bin. Gray or black bars indicate subjective day or night, respectively.

We identified 269 (Dataset 1: 3.3%), 157 (Dataset 2: 2.0%), and 297 transcripts (Dataset 3: 3.2%), whose APA index was rhythmic (Figure 2A-C, Table S1). We experimentally validated the 3′-end sequences of both proximal and distal poly(A) sites for selected genes by 3′- Rapid Amplification of cDNA ends (RACE) (Figure 2D-E). The 3′-end sequences were further verified by Sanger sequence and these matched with annotated 3′-end in the bioinformatical analyses (Hoque *et al.* 2013) (data not shown).

Eleven genes were found to be rhythmic in at least two datasets (*Ado*, *Cnot7*, *Cyhr1*, *Mapk8*, *Paqr9*, *Srsf10*, and *Tnfaip2* between Dataset 1 and 3, *Ccdc93*, *Ctbs*, and *Sdccag3* between Dataset 1 and 2, and *Hspa4* between Dataset 2 and 3) (Figure 2F). An additional 9 genes (*Magi1*, *Mrps5*, *Oxr1*, *Pcf11*, *Samhd1*, *Ssh1*, *Ttpal*, *Vsp26a*, *Zfp259*) had APA indexes that were rhythmic in two datasets; however, we did not categorize these genes as “overlapping” because, although one isoform was commonly detected in two datasets, the other isoform (typically the second highest ψ value) was not identical between the two datasets. Of note, we found one transcript (*Ccdc93*) that was represented by all three datasets when we slightly lowered the rhythmicity detection threshold (Table S1). Nevertheless, the small overlap between datasets is probably due to the differences in experimental conditions, such as the duration of sampling (24 hr for Dataset 2, and 48 hr for Dataset 1 and 3), the sampling interval (4 hr for Dataset 1, 3 hr for Dataset 2, and 6 hr for Dataset 3, the sampling start time (CT 0 for Datasets 1 and 2, and CT 22 for Dataset 3), and the source RNA used for library construction (ribosomal depleted RNA for Dataset 1 and poly(A)^+^ enriched RNA for Datasets 2 and 3), as well as technical noise over the course of experiments and analyses (Conesa *et al.* 2016).

One remarkable feature of APA is the global shortening or lengthening of transcripts (Flavell *et al.* 2008; Mayr and Bartel 2009). However, we did not detect any particular time points during which the APA index was disproportionally enriched (Figure 2G), although each dataset exhibited unique patterns (Supplemental Figure 1). This is in contrast to transcription and translation, both of which are under global regulation in mouse liver, peaking during early subjective night (Koike *et al.* 2012; Menet *et al.* 2012; Jouffe *et al.* 2013; Janich *et al.* 2015). This is also in stark contrast with circadian poly(A) tail length regulation, where the rhythmicity of poly(A) tail length peaks predominantly at subjective night (Kojima *et al.* 2012).

In order to shed light on the cellular processes that are regulated by circadian APA genes, we performed a gene ontology analyses using DAVID (Huang da *et al.* 2009a; Huang da *et al.* 2009b). The top 10 pathways/processes that were significantly enriched, were “Transcription,” “Nucleotide/ATP-binding,” “Metal(zinc)-binding,” “Endocytosis,” “Protein transport,” “Cell cycle,” “RNA-binding,” “Endoplasmic reticulum,” “Fibronectin type-II,” and “Biological rhythms” (Table S2). Notably, “Transcription” had the highest enrichment score in all three datasets (Table S2). It is possible that circadian APA indirectly regulates rhythmic transcription by affecting RNA stability or translation efficiency, for example, of those genes functioning in transcription.

### Canonical PASs at distal sites are more frequently used in mouse liver, although proximal poly(A) sites more frequently used among rhythmic APA transcripts

**Figure 3:**
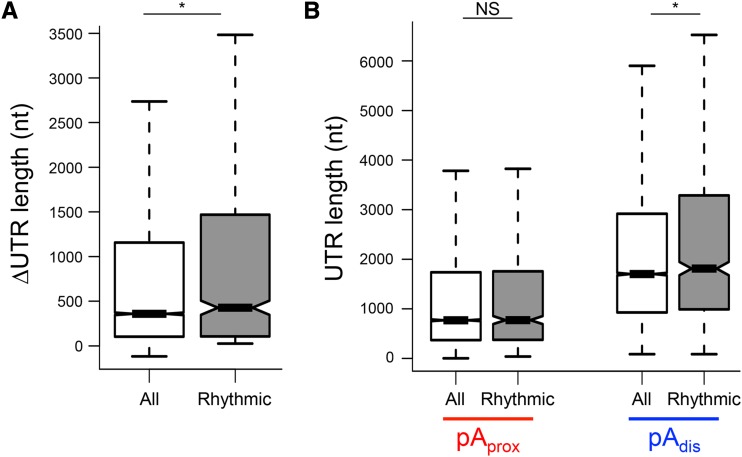
Rhythmic APA genes have longer 3′UTRs. (A) Difference in the 3′UTR length between APA transcripts. (B) Distributions of 3′UTR length of the short (*i.e.*, proximal poly(A) site usage) and long (*i.e.*, distal poly(A) site usage) isoforms. All: n = 15246, and rhythmic: n = 723. Box-whisker plots with quartiles (box) and quartile ± 1.5 interquartile range (whiskers). *; *P* < 0.05. (Mann-Whitney-Wilcoxon Test). pA_prox_: proximal poly(A) site, pA_dis_: distal poly(A) site.

To shed light into the mechanism of circadian APA regulation, we next characterized the relative distance, location, sequence, and frequency of alternative poly(A) sites, and compared these characteristics between rhythmic and all APA transcripts (control). In mouse liver, we found that the relative 3′UTR length difference between alternatively polyadenylated transcripts is longer among rhythmic APA transcripts (median: 427 nt) compared to control APA transcripts (median: 359 nt) (Mann-Whitney-Wilcoxon test: *P* = 0.02806) (Figure 3A). Interestingly, the 3′UTR length of short isoforms was not different between all (median: 768.5 nt) and rhythmic APA transcripts (median: 772 nt) (Figure 3B). In contrast, the 3′UTR length of long isoforms was increased in rhythmic APA (median: 1811 nt) compared to all APA transcripts (median:1700.5 nt) (Mann-Whitney-Wilcoxon test: *P* = 0.02052) (Figure 3B). These data suggest that the long isoforms of rhythmic APA transcripts contain more *cis*-regulatory elements and are more likely subject to post-transcriptional regulation.

The most frequently detected PAS is AAUAAA, accounting for ∼60% of PASs among all expressed cDNAs/ESTs in human and mouse (Beaudoing *et al.* 2000; Tian *et al.* 2005). Another approximately 20 PAS variants have been observed, albeit less frequently (ex., AUUAAA: ∼15%), while some transcripts do not contain an obvious PAS at all (Beaudoing *et al.* 2000; Tian *et al.* 2005). Our analysis revealed that 64.2% of single PAS genes contained the canonical signal, AAUAAA, and 17.7% contained the variant AUUAAA within 40 nt from the 3′-end of each transcript (Table 3). In contrast, genes possessing more than one PAS (*i.e.*, APA genes) had lower AAUAAA usage and only 43.2% possessed AAUAAA, whereas 17.4% contained AUUAAA (Table 3). The actual percentage may be even lower, since we counted the same motif twice if both poly(A) sites are within 40 nt of one another, which was the case for 7.9% of genes.

**Table 3:** APA transcripts and their PAS types

Dataset	Single PAS		Multiple PAS	
	AAUAAA (%)	AUUAAA (%)	AAUAAA (%)	AUUAAA (%)
Dataset1	1004 (60.6)	276 (16.7)	6380 (46.8)	2606 (17.9)
Dataset2	650 (64.8)	186 (18.6)	5330 (42.6)	2096 (16.8)
Dataset3	1364 (67.0)	363 (17.8)	6985 (42.6)	2762 (16.8)
**Combined**	3018 (64.2)	825 (17.6)	18695 (43.0)	7464 (17.2)

**Figure 4:**
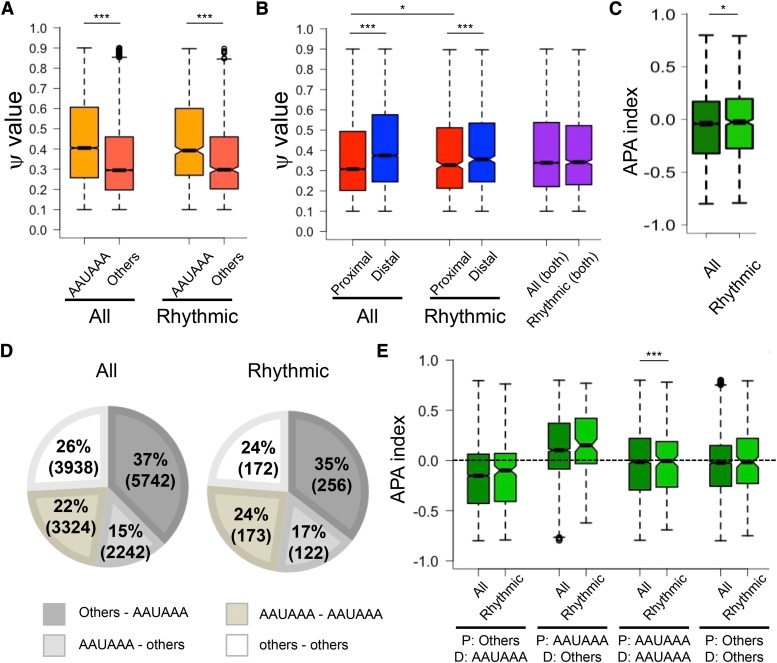
Hepatic APA genes use distal poly(A) sites and canonical PASs more frequently, but circadian APA genes have less distinct difference. (A) Distributions of ψ values in APA transcripts that have a canonical PAS (AAUAAA) or other PAS variants. All: AAUAAA; n = 14632, others; n = 15860, and rhythmic: AAUAAA; n = 724, others; n = 722. (B) Distributions of ψ values in transcripts that use distal or proximal poly(A) sites. All: proximal = 15246 and distal = 15246, Rhythmic: proximal = 723 and distal = 723. (C) Distribution of the APA index between all (N= 15246) and rhythmic (N= 723) APA transcripts. (D) Percentages of each PAS combination group. All: N = 15246, Rhythmic: N= 723. Numbers in parentheses indicate the number of transcripts that fall under each combination group. (E) Distribution of the APA index in each PAS group. All the box-whisker plots represent quartiles (box) and quartile ± 1.5 interquartile range (whiskers). *; *P* < 0.05. ***; *P* < 0.005 (Mann-Whitney-Wilcoxon Test).

The canonical PAS (AAUAAA) has been considered strongest among other variants, due to its high prevalence among cDNAs/ESTs (Beaudoing *et al.* 2000; Tian *et al.* 2005). By using the average ψ values across all time points as an indicator of poly(A) site strength, we found that the isoforms with canonical PASs were indeed more frequent in mouse liver (Figure 4A, S2A). The median ψ value for isoforms with AAUAAA was 0.41, whereas that of other variants was 0.30 in all APA transcripts (Mann-Whitney-Wilcoxon test: *P* < 2.2e-16) (Figure 4A, S2A). This trend was also observed among rhythmic APA transcripts, in which the median ψ value for isoforms with AAUAAA was 0.39, whereas that of other variants was 0.30 (Mann-Whitney-Wilcoxon test: *P* < 2.2e-16) (Figure 4A, S2A).

Each tissue has a different frequency for the poly(A) site usage, with the distal sites generally being more frequently used in non-proliferating cells (Hoffman *et al.* 2016), such as hepatocytes that make up ∼80% of the liver. We found that distal sites were stronger than proximal sites in mouse liver, as the median ψ value of distal sites was 0.38, while that of proximal sites was 0.31 among all APA transcripts (Mann-Whitney-Wilcoxon test: *P* < 2.2e-16) (Figure 4B, S2B). Distal sites were also stronger among rhythmic APA transcripts, as the median ψ value of distal sites was 0.36, while that of proximal sites was 0.33 (Mann-Whitney-Wilcoxon test: *P* < 2.2e-16). Interestingly, however, the difference between proximal and distal sites was smaller in rhythmic APA transcripts (Δ median ψ_distal-proximal_ = 0.03), compared to control APA transcripts (Δ median ψ_distal-proximal_ = 0.07). This was not due to differences in the distribution of ψ values between all and rhythmic APA transcripts, as the median ψ value that includes both proximal and distal sites were the same (median ψ = 0.39) for both all and rhythmic APA transcripts (Figure 4B, S2B). Rather, this was because rhythmic APA transcripts use proximal poly(A) sites more frequently, as the median ψ values of proximal sites higher among rhythmic APA transcripts (median: 0.33) compared to all APA transcripts (median: 0.31) (Mann-Whitney-Wilcoxon test: *P* = 0.02134). This was further supported through comparison of the APA index, which represents the relative strength between proximal and distal poly(A) sites. We found that rhythmic APA transcripts had slightly, but significantly, higher APA index (median: -0.025) compared to all APA transcripts (median: -0.04) (Mann-Whitney-Wilcoxon test: *P* = 0.02811) (Figure 4C), indicating that rhythmic APA transcripts use proximal poly(A) sites more frequently.

Genes that have more than one poly(A) site often have canonical PASs at the most distal site, while variants tend to be found at more proximal sites, despite that promoter-proximal poly(A) sites have a natural advantage over distal ones to be transcribed first and can be used more frequently (Beaudoing *et al.* 2000; Tian *et al.* 2005; Davis and Shi 2014). This was also the case in mouse liver, and transcripts with a variant at a proximal site and AAUAAA at a distal site were most frequent among both all and rhythmic APA transcripts (Figure 4D, S3A). In addition, rhythmic APA transcripts tended to have higher APA indices relative to all transcripts, regardless of the combination of PASs at proximal and distal poly(A) sites (Figure 4E, S3B), consistent with our earlier observation (Figure 4C).

These results demonstrate that canonical PAS (AAUAAA) and distal sites are most commonly used in mouse liver. Rhythmic APA genes, in contrast, have less distinct differences in poly(A) site strength between proximal and distal sites and use proximal sites more frequently. Presumably, this allows the daily poly(A) site switch to be more amenable.

## Discussion

In this study, we identified and characterized transcripts undergoing rhythmic alternative polyadenylation (APA) in mouse liver, using existing circadian transcriptome datasets combined with a probabilistic algorithm, MISO (Katz *et al.* 2010; Koike *et al.* 2012; Vollmers *et al.* 2012; Zhang *et al.* 2014). Identification of differentially expressed transcripts from transcriptome data has been a challenge, because distinguishing full-length isoforms from partially reconstructed fragments is not always possible and requires greater sequencing depth and more powerful statistical methods (Trapnell *et al.* 2009; Katz *et al.* 2010). MISO circumvents this issue by inverting the process by which reads are first produced and then inferring the underlying isoform abundances that best explain the observed reads. Our *in silico* analysis identified 712 transcripts (or 2.9% of all expressed genes) that undergo circadian APA regulation in mouse liver.

The number of circadian APA genes we detected in our analysis was smaller than a previous report, which identified 1153 circadian APA genes in mouse liver derived from microarray data (Liu *et al.* 2013). It is unclear how much our list of APA genes overlaps with the previous report, as the identity of these transcripts, except for a few, was not revealed (Liu *et al.* 2013). The difference in the number is most likely due to the frequency of tissue sampling, in which the liver samples were taken: every 1 hr (Hughes *et al.* 2009; Liu *et al.* 2013) or every 3-6 hr (this study). The other likely causes include the amplitude cutoff, the gene expression cutoff (*i.e.*, presence *vs.* absence), the rhythmicity detection algorithm, and quantification method of the APA switch (*i.e.*, “APA index” = ψ _proximal_ - ψ _distal_ *vs.* “the ext/com-ratio” (=ψ _distal_ / ψ _proximal_)). Most notably, our use of transcriptome data constitutes a significant advantage over that of microarray data, as it allowed us to pinpoint the exact 3′-end locations of each transcript and characterize features commonly found in circadian APA genes. Tiling arrays have higher probe density than conventional microarrays, and yet do not have the sensitivity and accuracy to distinguish isoforms, due to their probe length (∼25-60mer) and spacing (∼400-10,000 nt on average) (Liu 2007; Agarwal *et al.* 2010; Zhao *et al.* 2014). In addition, our use of three independent datasets alleviated observing dataset-specific effects (for example, Figure 2G, Supplemental Figure 1-3).

Although the mechanisms of the circadian APA are not fully understood, we found several interesting characteristics that are unique to rhythmic APA transcripts. One such characteristic is that rhythmic APA genes have less distinct differences in poly(A) site strength between proximal and distal sites (Figure 4), possibly due to the lower frequency of canonical PAS usage among rhythmic APA genes (Table 3). This presumably allows the daily poly(A) site switch to be more feasible. The most likely mechanism is that the *cis*-elements specifically found in the long isoform (*i.e.*, distal poly(A) site usage) either activate or repress one poly(A) site usage over another by interacting with *trans*-factors. It is also possible that these interactions affect the RNA processing (*i.e.*, mRNA stability, translation, and localization) (Di Giammartino *et al.* 2011; Mayr 2016). To support this, circadian APA transcripts have a larger difference in the 3′UTR length between the APA isoforms (Figure 3). Identifying these *cis*-elements and understanding how their interaction with *trans*-factors affect the target poly(A) site choice in a circadian manner will, thus, help us better understand the mechanisms circadian APA regulation in the future.

Since components of the cleavage and polyadenylation machinery, *e.g.*, CSTF77 and NUDT21, are expressed rhythmically in mouse liver (Mauvoisin *et al.* 2014; Robles *et al.* 2014), it is tempting to speculate that they are involved in regulating circadian APA at least for some genes. Changes in the levels of the other core components for cleavage and polyadenylation machinery, including CSTF-64/CSTG2, CSTF-64τ, CFI-68/CPSF6, CFI-25/CPSF5, FIP1L1, and PCF11 as well as auxiliary factors such as PABPN1, PTBP, RBBP6, and PAF1C, have all been shown to impact poly(A) site choices (Takagaki *et al.* 1996; Takagaki and Manley 1998; Kubo *et al.* 2006; de Klerk *et al.* 2012; Jenal *et al.* 2012; Martin *et al.* 2012; Yao *et al.* 2012; Yao *et al.* 2013; Di Giammartino *et al.* 2014; Lackford *et al.* 2014; Masamha *et al.* 2014; Gennarino *et al.* 2015; Li *et al.* 2015; Domingues *et al.* 2016; Yang *et al.* 2016). These proteins may also be involved in regulating circadian APA, although this remains to be investigated.

Despite the advances of sequencing technology and analytical tools that expand our ability to detect APA events, consequences of APA are still largely unknown. We measured the correlation between protein rhythmicity *vs.* APA rhythmicity utilizing three circadian proteome datasets from mouse liver (Reddy *et al.* 2006; Mauvoisin *et al.* 2014; Robles *et al.* 2014). We found that the percentage of rhythmic proteins was similar among circadian APA genes and control APA genes (data not shown). It is unclear whether this suggests that rhythmic APA regulation does not contribute to rhythmic protein production or the correlations are missed simply because the existing mammalian liver proteome data only cover a small percentage of the entire proteome. It is also possible that the impact of APA on translation is regulated by gene-specific, rather than global, mechanisms. Nevertheless, it would be of great interest in the future to examine the impact of circadian APA regulation on the proteome and/or transcriptome.

Obviously, experimental validation is key to underscoring the importance of our findings. Our attempts to measure circadian changes in APA by quantitative PCR were unsuccessful; however, quantitative PCR may not be an ideal method, since it is technically impossible to specifically detect shorter isoforms. Several genome-wide approaches are now available to detect 3′-end of transcripts quantitatively (Pelechano *et al.* 2012; Cornett and Lutz 2014; Hoque *et al.* 2014; Yao and Shi 2014; Xing and Li 2015; Zheng *et al.* 2016; Zheng and Tian 2017). These tools can be used in the future to quantify circadian APA, including *de novo* poly(A) sites.

Overall, our study provides evidence that the choice of poly(A) site and therefore mRNA 3′-end structure are under circadian regulation. Interesting research directions in the future would be to 1) experimentally validate the circadian poly(A) site switches, 2) identify *cis*-elements enriched in the circadian APA transcripts, and 3) delineate how these *cis*-elements confer rhythmicity in poly(A) site choices. By identifying unique characteristics of rhythmic APA genes and illustrating the landscape of poly(A) tails in mouse liver, our study serves as a platform to help us understand the mechanisms of circadian APA regulation.
